# Thermoreversible Gel-Dispersed Liquid Crystals

**DOI:** 10.3390/gels9120965

**Published:** 2023-12-08

**Authors:** Akihiko Matsuyama

**Affiliations:** Department of Physics and Information Technology, Faculty of Computer Science and Systems Engineering, Kyushu Institute of Technology, Kawazu 680-4, Iizuka 820-8502, Fukuoka, Japan; mazyama@phys.kyutech.ac.jp

**Keywords:** thermoreversible gel, liquid crystal, sol–gel transition, nematic–isotropic transition, phase separation

## Abstract

A simple model is introduced to describe phase behaviours of binary mixtures of a thermoreversible gel and a low-molecular-weight liquid crystal (LC). We predict novel phase diagrams on the temperature–concentration plane, including sol–gel transition, nematic–isotropic phase transition, and phase separation. At high temperatures, the phase separation between the isotropic sol and gel phases appears. As the temperature decreases, we have the phase separation between nematic sol and isotropic gel phases, in which the nematic domains are dispersed in the isotropic gel phase. We suggest that thermoreversible gelation of reactive molecules mixed with LCs will become one of the new classes of polymer-dispersed liquid crystals.

## 1. Introduction

Polymer-dispersed liquid crystals (PDLCs) are important to liquid crystal technology, such as displays, switchable windows, light shutters, etc. [1,2]. In these systems, nematic LC microdroplets are dispersed in a polymer matrix by using the phase separation between isotropic polymer-rich and nematic LC-rich phases [3,4,5,6,7,8,9,10,11]. Typical examples of LC/polymer mixtures are mixtures of (p-ethoxybenzylidene)-p-n-butylaniline (EBBA) and polystyrene (PS) [3] and mixtures of 4-cyano-4${}^{\prime }$-n-heptylbiphenyl (7CB) and PS [7], etc. PDLC films are formed by polymer compositions with LCs or mixtures of reactive monomers with LCs followed by their photopolymerisation [1]. The photocrosslinking reaction of the matrix polymer induces phase separation while the fluid mixture is hardened. The coexistence curve of an LC/monomer mixture has an upper critical solution temperature (UCST), and below UCST, phase separation between two isotropic phases appears. As polymerisation proceeds, the coexistence curve moves to higher temperatures towards the higher LC concentrations. The coexistence curve of an LC/crosslinked polymer mixture makes an upward turn near the pure LC axis on the temperature–concentration plane. When the coexistence curve crosses a reaction temperature, the system becomes thermodynamically unstable and undergoes phase separations [12]. These polymerisation-induced PDLCs are irreversible processes, meaning the polymer networks can not be broken by temperature changes.

On the other hand, sol–gel transitions are thermoreversible processes [13,14,15]. For example, intermolecular hydrogen bonding causes thermoreversible gelation in liquid crystalline systems [16,17]. The physical gelation has also been observed in atactic polystyrene (at-PS) mixed with the solvent molecule of carbon disulfide (CS${}_{2}$), in which the sol–gel transition and phase separations with UCST have been theoretically and experimentally studied [18,19,20,21,22,23,24]. The chemical structure of the chains, in particular the side group of the chain, has a significant influence on the gelation [24]. Thus, by using thermoreversible gelation of reactive molecules mixed with LCs, we can control LC/monomer and LC/network polymer mixtures by changing the temperature.

In this paper, we theoretically explore temperature-sensitive PDLCs formed by thermoreversible gels and LCs. We introduce a simple model to describe the phase behaviours of binary mixtures of a thermoreversible gel and an LC. Based on the molecular-field theories for nematic ordering of PDLCs [8,9,10,11] and for thermoreversible gelation [21,22,23], we construct the free energy of binary mixtures of reactive molecules and LCs. We calculate phase diagrams on the temperature–concentration plane and predict novel phase behaviours, such as phase separations between nematic sol and isotropic gel phases, caused by three different phase transitions: sol–gel transition, nematic–isotropic phase transition (NIT), and phase separation. We also discuss the phase behaviours of the mixture of an at-PS and LC. In Section 2, we introduce a molecular-field theory to describe the phase behaviours of the mixtures. In Section 3, we show some numerical results.

## 2. Theoretical Method

Let us consider binary mixtures of a thermoreversible reactive molecule and an LC. The functional groups on a reactive molecule are assumed to be identical and capable of forming physical bonds by pairwise association. The bonding energy considered here is of the order of thermal energy, and then the bonding–unbinding equilibrium is established by temperature changes. For example, hydrogen (physical) bonding becomes energetically stable at lower temperatures. In thermal equilibrium, the intermolecular bonding yields polydisperse molecular aggregates, and we call them ’*m*-cluster’ in the following, where m(=1,2,⋯,∞) is the number of reactive molecules on the cluster. To derive the size distribution of such clusters, we consider the thermodynamics of the system.

The free energy of our system can be constructed by the sum of three terms:

(1) $$F=F_{rea}+F_{mix}+F_{nem}.$$

The first term is the free energy change to form the clusters from the reference state, where pure LC solvent and *m*-clusters are separately prepared by pairwise connections of the functional group on the reactive molecules. It is given by

(2) $$F_{rea}=\sum_{m=1}^{\infty }N_{m}\mu_{m}^{\circ },$$

in terms of the chemical potential $\mu_{m}^{\circ }$ of a pure *m*-cluster, where $N_{m}$ is the number of the *m*-cluster. The second term $F_{mix}$ shows the mixing free energy change required in the process of mixing *m*-clusters and LCs. According to the Flory–Huggins theory for polymer blends, it is given by [22,23,25]

(3) $$\beta F_{mix}=N_{t}\left[\frac{\phi_{r}}{n_{r}}\ln\phi_{r}+\sum_{m=1}^{\infty }\frac{\phi_{m}}{nm}\ln\phi_{m}+\chi \phi_{r}\left(1-\phi_{r}\right)\right],$$

where $\phi_{r}\left(=n_{r}N_{r}/N_{t}\right)$ is the volume fraction of the LCs with the number $N_{r}$ of the molecules, $\phi_{m}\left(=nmN_{m}/N_{t}\right)$ is the volume fraction of the *m*-clusters, $\beta =1/(k_{B}T)$: $k_{B}$ is the Boltzmann constant and *T* is the absolute temperature, $N_{t}\left(=n_{r}N_{r}+n\sum_{m=1}^{\infty }mN_{m}\right)$ is the total number of lattice cells in the system, *n* is the number of segments on the reactive molecule, $n_{r}$ is the axial ratio of the LC, and χ is the Flory–Huggins interaction parameter between the reactive molecule and LC. The volume of the system is given by $V=a^{3}N_{t}$, where $a^{3}$ is the volume of a segment. The volume of the reactive molecule is given by $a^{3}n$, and the volume of the LC molecule is given by $a^{3}n_{r}$. This is a simple picture based on the lattice model of Flory–Hugggins’ theory. The total volume fraction of the reactive molecules is given by $\phi =\sum_{m=1}^{\infty }\phi_{m}$ and we have $\phi_{r}+\phi =1$. The third term in Equation (1) is the free energy for nematic ordering of LCs. Using the Maier–Saupe model for nematic ordering [10,11,26], it is given by

(4) $$\beta F_{nem}=N_{t}\left[\frac{\phi_{r}}{n_{r}}\int f_{d}\left(\theta \right)\ln4\pi f_{d}\left(\theta \right)d\Omega -\frac{1}{2}\nu \phi_{r}^{2}S^{2}\right],$$

where dΩ=2πsinθdθ is the solid angle, $f_{d}\left(\theta \right)$ is the orientational distribution function of LCs, and we have the normalisation condition:

(5) $$\int f_{d}\left(\theta \right)d\Omega =1.$$

We define here the dimensionless anisotropic parameter $\nu (=\beta U_{a})>0$, where $U_{a}$ is the orientation-dependent (Maier–Saupe) interaction between LCs. The orientational order parameter *S* of the LCs can be calculated by

(6) $$S=\int P_{2}\left(\cos\theta \right)f_{d}\left(\theta \right)d\Omega ,$$

where $P_{2}\left(x\right)=\left(3/2\right)x^{2}-1/2$ shows the second Legendre polynomial.

The orientational distribution function $f_{d}\left(\theta \right)$ is determined by minimising the free energy (Equation (4)) with respect to this distribution function. We then obtain (see Appendix A)

(7) $$f_{d}\left(\theta \right)=\frac{1}{Z_{0}}\exp\left[\Gamma SP_{2}\left(\cos\theta \right)\right],$$

where

(8) $$\Gamma =n_{r}\nu \phi_{r},$$

shows the strength of nematic ordering. Substituting Equation (7) into Equation (5), the normalisation constant $Z_{0}$ is given by $Z_{0}=4\pi I_{0}\left(\Gamma S\right)$, where the function $I_{0}$ is defined as

(9) $$I_{q}\left(\Gamma S\right)=\int_{0}^{1}{\left(P_{2}\left(x\right)\right)}^{q}\exp(\Gamma SP_{2}\left(x\right))dx,$$

where q=0,1 and x=cosθ. Substituting Equation (7) into Equation (6), the orientational order parameter of the LCs is determined by the self-consistent equation

(10) $$S=I_{1}\left(\Gamma S\right)/I_{0}\left(\Gamma S\right).$$

Using Equation (7), the nematic free energy (Equation (4)) is given by

(11) $$\beta F_{nem}/N_{t}=\frac{1}{2}\nu \phi_{r}^{2}S^{2}-\frac{\phi_{r}}{n_{r}}\ln I_{0}\left(\Gamma S\right).$$

### 2.1. Sol–Gel Transition

In thermal equilibrium, each reactive molecule is in chemical equilibrium through bonding and unbonding processes. This imposes the following multiple-equilibria condition [23]:

(12) $$m\mu_{1}=\mu_{m},$$

where $\mu_{m}={\left(\partial F/\partial N_{m}\right)}_{N_{r}}$ is the chemical potential of the *m*-cluster:

(13) $$\begin{array}{ccc} \beta \mu_{m} & = & \beta \mu_{m}^{\circ }+\ln\phi_{m}+1-\frac{nm}{n_{r}}+nm\left(\frac{\phi }{n_{r}}-\frac{\sum \phi_{m}}{n\langle m\rangle }\right)+nm\chi {\left(1-\phi \right)}^{2}+\frac{1}{2}\nu \phi_{r}^{2}S^{2}, \end{array}$$

$\mu_{1}$ is the chemical potential of the monomer (*m* = 1), and

(14) $$\langle m\rangle =\sum_{m=1}^{\infty }\phi_{m}/\sum_{m=1}^{\infty }\left(\phi_{m}/m\right),$$

is the number-average mean cluster size of *m*-clusters. We also have the chemical potential of the LCs $\mu_{r}={\left(\partial F/\partial N_{r}\right)}_{N_{m}}$:

(15) $$\beta \mu_{r}=\beta \mu_{r}^{\circ }+\ln\left(1-\phi \right)+\phi -\frac{n_{r}\sum \phi_{m}}{n\langle m\rangle }+n_{r}\chi \phi^{2}+\frac{1}{2}n_{r}\nu S^{2}\phi^{2}-\ln I_{0}\left(\Gamma S\right).$$

where $\mu_{r}^{\circ }$ is the chemical potential of a pure LC. Substituting the chemical potentials (Equation (13)) into Equation (12), we obtain the volume fraction of the *m*-clusters:

(16) $$\phi_{m}=K_{m}\phi_{1}^{m},$$

where the association constant $K_{m}$ is given by

(17) $$K_{m}=\exp\left(-(\delta_{m}+\frac{1}{m}-1)m\right),$$

in terms of the dimensionless free energy difference $\delta_{m}$ for the *m*-cluster formation:

(18) $$\delta_{m}=\beta \left(\mu_{m}^{\circ }-m\mu_{1}^{\circ }\right)/m.$$

Within the radius of convergence, the equation $\phi =\sum \phi_{m}$ gives a one-to-one relationship between the total volume fraction ϕ and the volume fraction of monomer $\phi_{1}$. By inverting this relation, the monomer concentration $\phi_{1}$ is given as a function of the total concentration ϕ: $\phi_{1}\left(\phi \right)$, for $0\leq \phi_{1}\leq \phi$.

We can define sol or gel states depending on the function $\delta_{m}$ [21]. Let $m^{*}$ be the cluster size at which the volume fraction $\phi_{m}$ takes a maximum for a given $\phi_{1}$. Since the value of $\phi_{m}$ does not exceed unity by definition, the monomer concentration $\phi_{1}$ is limited by the inequality $K_{m^{*}}\phi_{1}^{m^{*}}\leq 1$ and hence we have

(19) $$\phi_{1}\leq \phi_{1}^{*}=\exp\left(\delta_{m^{*}}+1/m^{*}-1\right).$$

When $m^{*}$ is finite, the upper bound of Equation (19) is called “critical micelle concentration”. Sharpness in the appearance of the clusters is controlled by the curvature of the function $\delta_{m^{*}}+1/m^{*}-1$ around $m^{*}$. If the function $\delta_{m}$ is a monotonous decrease function of *m*, the cluster size $m^{*}$ becomes infinite, and a macroscopic cluster (gelation) appears at $\phi_{1}^{*}=\exp\left(\delta_{\infty }-1\right)$. We then have a sol–gel transition at the total volume fraction $\phi^{*}$ obtained from $\phi_{1}^{*}$. The summation of the power series $\sum \phi_{m}$ does not reach ϕ for $\phi_{1}$ above $\phi_{1}^{*}$ because it can not include the contribution from the infinite network $\phi_{\infty }$. Then the excess volume fraction $\phi -\sum \phi_{m}$ corresponds to the gel component $\phi_{G}$ and $\phi_{S}=\sum \phi_{m}$ is the sol component: $\phi =\phi_{S}+\phi_{G}$. The chemical potential $\mu_{G}$ of a single molecule participating in the gel network can be found by $\mu_{m}/m$ for m→∞, and we have an additional condition $\mu_{G}=\mu_{1}$ for the molecular association. The unimer concentration remains fixed at $\phi_{1}^{*}$ above the gelation threshold, while the total concentration increases. This is a simple picture of gelation based on the mean-field approximation [27,28].

To proceed a step further, we here consider a model for the internal structure of a cluster in the form of the Kayley tree of *f*-functional molecules, where the intracluster loop formation is neglected. This is a simple approximation based on the classical theory for gelation. The internal partition function $Z_{m}$ of a single *m* cluster can be given by [28]

(20) $$Z_{m}=\left(W_{m}\left(f\right)/m!\right)p^{m-1}{\left(1-p\right)}^{fm-2m+2},$$

where *p* is the probability of bond formation for a pair of active groups and $W_{m}\left(f\right)$ is the number of combinations in which *m* molecules form a tree. This combinatorial factor is given by $W_{m}\left(f\right)=\left(fm-m\right)!f^{m}/\left(fm-2m+2\right)!$, provided the *f*-functional groups are indistinguishable [27]. The partition function $Z_{m}$ gives the free energy change $\delta_{m}$ through the relation $\exp\left(-m\delta_{m}\right)=Z_{m}/Z_{1}^{m}$, by definition. Then, the volume fraction of the *m*-cluster is given by

(21) $$\phi_{m}=\frac{{\left(1-p\right)}^{2}}{pe}\frac{(fm-m)!}{m!(fm-2m+2)!}{\left(\lambda \phi_{1}\right)}^{m},$$

where we define the parameter $\lambda =efp/{\left(1-p\right)}^{2}$. According to the general prescription, the infinite series $\sum \phi_{m}$ can be explicitly summed up by introducing a parameter α defined by $\lambda \phi_{1}=\alpha {\left(1-\alpha \right)}^{f-2}$. We find $\lambda \phi =\alpha \left(1-f\alpha \right)/{\left(1-\alpha \right)}^{2}$ within the radius of convergence [27,28]. The limit of convergence is given by $\alpha_{c}=1/\left(f-1\right)$, or equivalently, (see Appendix B)

(22) $$\phi^{*}=\frac{1}{2(f-2)\lambda }.$$

This equation shows the sol–gel transition. In our molecular-field theory, the reversible gelation is the second-order phase transition, where the osmotic pressure has a kink at the sol–gel transition point as a function of concentration ϕ [22]. The statistical weight λ of a bond formation relative to the weight of two unbounded functionalities is expressed as $p/{\left(1-p\right)}^{2}=\exp\left(-\beta \Delta f_{0}\right)$ in terms of the free energy change $\Delta f_{0}$ of a single bond formation. Splitting the free energy into the entropy Δs and energy Δϵ for the physical bond formation, $\Delta f_{0}=\Delta \epsilon -T\Delta s$, the parameter λ is given as a function of temperature, $\lambda \left(T\right)=\lambda_{0}\exp\left(-\beta \Delta \epsilon \right)$, where $\lambda_{0}=ef\exp\left(\Delta s/k_{B}\right)$ is the entropy parameter. Then, we can calculate the sol–gel transition line from Equation (22) on the temperature–concentration plane.

### 2.2. Nematic–Isotropic Phase Transition

**Figure 1:**
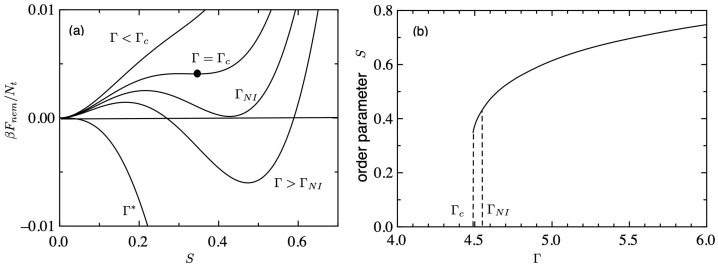
(**a**) Nematic free energy ($\beta F_{nem}/N_{t}$) of Equation (11) plotted against the order parameter *S* for various values of $\Gamma (=n_{r}\nu \phi_{r}$). (**b**) Orientational order parameter *S* plotted against Γ.

Figure 1a shows the nematic free energy ($\beta F_{nem}/N_{t}$) of Equation (11) plotted against the order parameter *S* for various values of Γ. For lower values of $\Gamma <\Gamma_{c}\left(≃4.49\right)$, the free energy has a single minimum at S=0, which corresponds to an isotropic phase. The closed circle shows a critical point (CP) at $\Gamma =\Gamma_{c}$. When Γ exceeds the critical value $\Gamma_{c}$, another minimum appears at a positive *S*, which corresponds to a metastable nematic phase. The first-order phase transition from an isotropic to a nematic phase takes place at

(23) $$\Gamma_{NI}=n_{r}\nu \phi_{r}≃4.55,$$

where the free energy of the isotropic phase becomes equal to that of the nematic phase. For intermediate values $\Gamma_{NI}<\Gamma <\Gamma^{*}\left(=5\right)$, two minima are observed: one (S=0) is metastable, and the other (S>0) shows a stable state. For larger values of $\Gamma >\Gamma^{*}$, the free energy has a single minimum at S>0, which corresponds to the nematic phase.

Figure 1b shows the order parameter *S* plotted against Γ. The first-order NIT occurs at $\Gamma_{NI}$, where the orientational order parameter *S* jumps from 0 to 0.44. The value of the order parameter increases from 0.44 to 1 with increasing Γ. Using Equation (8), the volume fraction at which the NIT takes place is given by $\phi_{r,NI}=4.55/\left(n_{r}\nu \right)$. Between $\Gamma_{c}<\Gamma <\Gamma_{NI}$, the system corresponds to a metastable region and the pre-transitional behaviours have been observed [29]. In this region, we have various anomalies in the physical properties such as a steep increase of the birefringence, and a slowing down of the relaxation process [30].

### 2.3. Solution Properties

Due to the Gibbs–Duhem relation for multi-component systems, the free energy (Equation (1)) can be expressed as

(24) $$F=N_{t}\left(\frac{\mu_{r}}{n_{r}}\phi_{r}+\sum_{m=1}^{\infty }\frac{\mu_{m}}{nm}\phi_{m}\right).$$

Using the chemical equilibrium condition (Equation (12)), the free energy can be written as

(25) $$F=N_{t}\left(\frac{\mu_{r}}{n_{r}}\phi_{r}+\sum_{m=1}^{\infty }\frac{\mu_{1}}{n}\phi_{m}\right)=N_{t}\left(\frac{\mu_{r}}{n_{r}}\phi_{r}+\frac{\mu_{1}}{n}\phi \right),$$

and results in the free energy of binary mixtures. The chemical potentials $\mu_{1}$ and $\mu_{r}$ are given by Equation (13) and Equation (15), respectively. Then, the free energy difference from that $F_{ref}$ of the reference state, where pure solvent and unreacted molecules are separately prepared, is given by

(26) $$\Delta F=F-F_{ref}=N_{t}\left(\frac{\Delta \mu_{r}}{n_{r}}\phi_{r}+\frac{\Delta \mu_{1}}{n}\phi \right),$$

where we define $\Delta \mu_{r}=\mu_{r}-\mu_{r}^{\circ }$ and $\Delta \mu_{1}=\mu_{1}-\mu_{1}^{\circ }$. Substituting Equations (13) and (15) into Equation (26), we obtain

(27) $$\begin{array}{ccc} \beta \Delta F/N_{t} & = & \frac{1-\phi }{n_{r}}\ln\left(1-\phi \right)+\frac{\phi }{n}\ln\phi_{1}+\chi \phi \left(1-\phi \right)+\frac{1}{n}\left(\phi -\frac{\phi_{S}}{\langle m\rangle }\right)\\ & & +\frac{1}{2}\nu S^{2}\phi_{r}^{2}-\frac{\phi_{r}}{n_{r}}\ln I_{0}\left(\Gamma S\right). \end{array}$$

The value of the $\phi_{1}$ is given as a function of ϕ for the sol region ($\phi \leq \phi^{*}$), however, becomes a constant $\phi_{1}^{*}$ for the gel region ($\phi >\phi^{*}$). The value of 〈m〉 also becomes a constant ${\langle m\rangle }^{*}$ for the gel region.

The conditions of binodal curves for two-phase coexistence are given by

(28) $$\mu_{r}\left(\phi^{\prime }\right)=\mu_{r}\left(\phi^{\prime \prime }\right),$$

and

(29) $$\mu_{m}\left(\phi^{\prime }\right)=\mu_{m}\left(\phi^{\prime \prime }\right),$$

for m=1,2,⋯∞, where the concentration $\phi^{\prime }$ and $\phi^{\prime \prime }$ show the volume fraction of reactive molecules in lower and higher concentrations in two coexisting phases, respectively. Using the chemical equilibrium condition (Equation (12)), Equation (29) results in

(30) $$\mu_{1}\left(\phi^{\prime }\right)=\mu_{1}\left(\phi^{\prime \prime }\right).$$

The coupled equations above (Equations (28) and (30)) correspond to the common tangent method, namely the free energy has a common tangent at $\phi^{\prime }$ and $\phi^{\prime \prime }$ [11].

The metastable and unstable regions on the temperature–concentration plane are separated by spinodal curve, ${\left(\partial \mu_{r}/\partial \phi \right)}_{T}=0$, which corresponds to the inflexion points of the free energy ${\left(\partial^{2}\left(F/N_{t}\right)/\partial \phi^{2}\right)}_{T}=0$. This leads to

(31) $$\frac{1}{1-\phi }-2\chi n_{r}+\frac{n_{r}\kappa \left(\phi \right)}{n\phi }-n_{r}\nu S^{2}=0,$$

where $\kappa \left(\phi \right)=\phi d\ln\phi_{1}\left(\phi \right)/d\phi$ for $\phi <\phi^{*}$ and κ(ϕ)=0 for $\phi >\phi^{*}$ as a function of ϕ.

The number-average mean cluster size (Equation (20)) for $\phi <\phi^{*}$ is given by

(32) $$\langle m\rangle =\sum_{m=1}^{\infty }mN_{m}/\sum_{m=1}^{\infty }N_{m}=\sum_{m}^{\infty }\phi_{m}/\sum_{m}^{\infty }\left(\frac{\phi_{m}}{m}\right)=\phi /\int_{0}^{\phi }\kappa \left(\phi \right)d\phi ,$$

and becomes a constant for $\phi^{*}\leq \phi$:

(33) $${\langle m\rangle }^{*}=\phi_{S}/\int_{0}^{\phi_{S}}\kappa \left(\phi \right)d\phi .$$

## 3. Results and Discussion

In this section, we show some numerical results of phase diagrams. For the numerical calculations, we define the reduced-temperature τ=1/χ. Using Equation (8), we obtain the NIT temperature as a function of the volume fraction of the reactive molecules,

(34) $$\tau_{NI}=n_{r}\alpha_{N}\left(1-\phi \right)/4.55,$$

where we define the dimensionless nematic parameter $\alpha_{N}=\nu /\chi$ [10]. The smaller values of $\alpha_{N}$ correspond to larger values of the χ parameter, and the solubility between solute and solvent molecules becomes poorer. When $\tau <\tau_{NI}$, the nematic phase is stable and when $\tau >\tau_{NI}$, the isotropic phase is stable. The orientational order parameter *S* jumps from 0 to 0.44 at $\tau =\tau_{NI}$ [11]. For the pure LCs (ϕ=0), the NIT temperature is given by $\tau_{NI}^{\circ }=n_{r}\alpha_{N}/4.55$. It is convenient to introduce the temperature parameter $\tilde{T}\left(=T/T_{NI}^{\circ }=\tau /\tau_{NI}^{\circ }\right)$ divided by the NIT temperature $T_{NI}^{\circ }$, or $\tau_{NI}^{\circ }$, and then the NIT temperature is given by

(35) $${\tilde{T}}_{NI}=T_{NI}/T_{NI}^{\circ }=\tau_{NI}/\tau_{NI}^{\circ }=1-\phi .$$

Note that the NIT temperature ${\tilde{T}}_{NI}$ is the universal function of ϕ for any LC, which does not depend on molecular characteristics such as $n_{r}$ and ν. We also define the dimensionless boding energy parameter $\gamma_{1}=-\Delta \epsilon /\left(k_{B}T_{NI}^{\circ }\right)$ and then we have $\lambda \left(T\right)=\lambda_{o}\exp\left(\gamma_{1}/\tilde{T}\right)$. Using Equation (22), the sol–gel transition temperature $T_{SG}$ is given by

(36) $${\tilde{T}}_{SG}=T_{SG}/T_{NI}^{\circ }=\frac{-\gamma_{1}}{\ln\left(2\left(f-2\right)\lambda_{0}\phi \right)},$$

as a function of ϕ. (Note that $\ln\left(2\left(f-2\right)\lambda_{0}\phi \right)<0$). On increasing the value of the bonding energy parameter $\gamma_{1}$, the temperature ${\tilde{T}}_{SG}$ increases higher.

**Figure 2:**
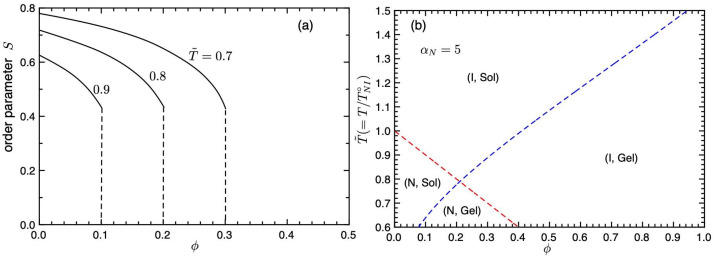
(**a**) Orientational order parameter *S* plotted against ϕ for $\tilde{T}=0.7,0.8,0.9$. (**b**) Phase transition curves on the temperature–concentration plane. The red broken line is the NIT (Equation (35)) and the blue broken line shows the sol–gel transition line (Equation (36)).

In the following, we take n=1, $n_{r}=2$, f=3, $\gamma_{1}=2.5$, and $\lambda_{0}=0.1$ for a typical example. Figure 2a shows the orientational order parameter *S* plotted against the concentration ϕ for various values of $\tilde{T}$. The orientational order parameter *S* jumps from 0 to 0.44 at the NIT, which shows the first-order phase transition. The value of *S* increases with decreasing concentration ϕ and temperature $\tilde{T}$. Figure 2b shows phase transition curves on the temperature–concentration plane. The red broken line is the first-order NIT (Equation (35)) and the blue broken line is the second-order sol–gel transition line (Equation (36)). The concentration $\phi_{NI}^{*}$ of the intersection between NIT and sol–gel lines is given by ${\tilde{T}}_{NI}={\tilde{T}}_{SG}$, which satisfies

(37) $$\left(1-\phi_{NI}^{*}\right)\ln\left(2\left(f-2\right)\lambda_{0}\phi_{NI}^{*}\right)=-\gamma_{1},$$

and we obtain $\phi_{NI}^{*}≃0.21$ and $T_{NI}^{*}≃0.79$. We find four different regions. The region (I, Sol) above the NIT and sol–gel lines shows the isotropic sol phase, (I, Gel) is the isotropic gel phase, and (N, Sol) shows the nematic sol state. The (N, Gel) region shows the nematic gel phase, where the nematic LCs are dispersed in the isotropic gel. Because the concentration of LCs is high and we have large values of the order parameter, S≃0.8 (see Figure 2a), the whole system seems to be nematic gel. Due to these regions, we have various phase separations. However, the intersection disappears inside the coexistence curves, as shown below.

**Figure 3:**
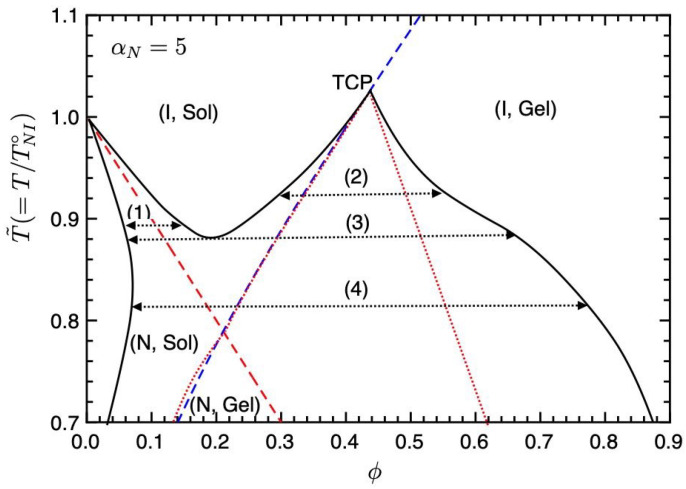
Phase diagram on the temperature–concentration plane for $\alpha_{N}=5$. The solid curve shows the binodal line, the red broken line is the NIT (Equation (35)), and the blue broken line is the sol–gel transition (Equation (36)). The dotted lines show the spinodal curve below which corresponds to the unstable region. We have four different phase separations (1)–(4). See text for details.

Figure 3 shows the phase diagram on the temperature–concentration plane for $\alpha_{N}=5$. The solid curve shows the binodal line (coexistence curve), the red broken line is the NIT (Equation (35)), and the blue broken line is the sol–gel transition line (Equation (36)). The dotted lines show the spinodal curve below which corresponds to unstable regions. The region between the spinodal and binodal lines shows metastable states. A part of the spinodal curve overlaps with the sol–gel line. On the phase diagram, we have four different regions (I, Sol), (I, Gel), (N, Sol), and (N, Gel). Due to these regions, we find four phase separations (1)–(4). Below the NIT temperature $\tilde{T}<1$, we have the phase separation (1) between the nematic and isotropic phases, observed in typical PDLC systems, such as mixtures of 7CB (LC) and PS, etc., [3,7]. In region (2), the phase separation between the isotropic sol and gel phases occurs. In our molecular-field theory, the reversible gelation is the second-order phase transition; therefore, the point where the gelation line meets the binodal at the top of the unstable region shows a tricritical point (TCP) [22]. On increasing the value of $\alpha_{N}$, or decreasing the χ parameter, the TCP shifts to lower concentrations and the phase separation (2) disappears. The intersection between the first-order NIT and sol–gel transition lines also shows TCP; however, this TCP is hidden inside the coexistence curves. We also find the triple point (TP) (3) where the nematic sol, isotropic sol, and isotropic gel phases coexist. Below the TP, we have the broad biphasic region between the nematic sol and isotropic gel phases (4), where the nematic phase consists of almost pure LCs and the isotropic gel phase consists of isotropic LCs and networks. In region (4), the nematic domains of LCs are dispersed in the isotropic gel. We predict that temperature changes can control these phase behaviours: (1)↔(3)↔(4) and (2)↔(3)↔(4).

**Figure 4:**
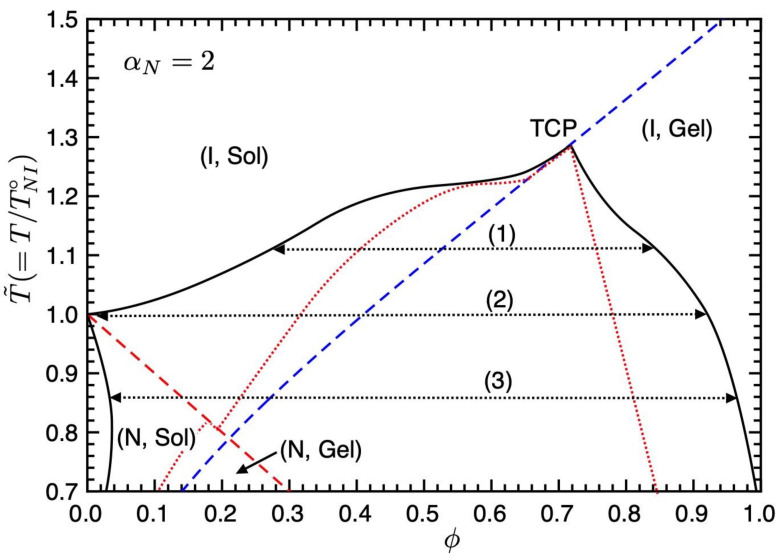
Phase diagram on the temperature–concentration plane for $\alpha_{N}=2.0$. The solid curve shows the binodal line, the red broken line is the NIT (Equation (35)), and the blue broken line is the sol–gel transition (Equation (36)). The dotted lines show the spinodal curve below which corresponds to the unstable region. We have three different phase separations (1)–(3). See text for details.

Figure 4 shows the phase diagram on the temperature–concentration plane for $\alpha_{N}=2.0$. On decreasing the value of the nematic parameter $a_{N}\left(=\nu /\chi \right)$, meaning the value of the χ-parameter increases [10], the binodal curve in the (I, Sol) region shifts upward, and we have three different phase separations (1)–(3). In region (1), we have the phase separation between the isotropic sol and gel phases. At the NIT temperature (2), the phase separation between the pure LC phase, where the I and N sol phases coexist, and the isotropic gel phase appears. Below the NIT temperature, we have the broad biphasic region between the nematic sol and isotropic gel phases (3), which appears in Figure 3.

**Figure 5:**
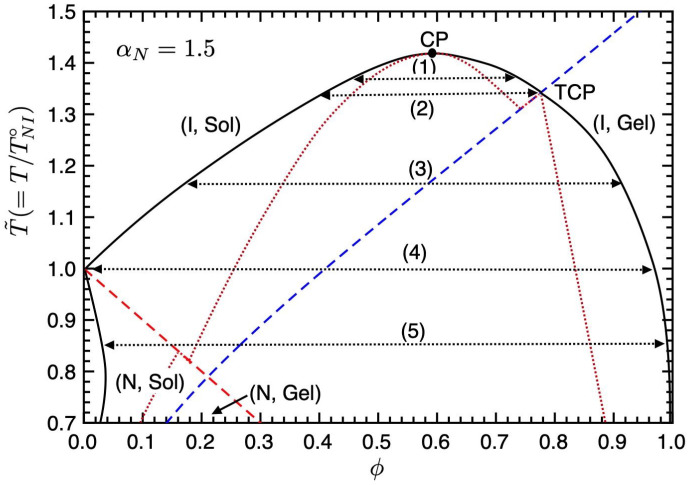
Phase diagram on the temperature–concentration plane for $\alpha_{N}=1.5$. The solid curve shows the binodal line, the red broken line is the NIT (Equation (35)), and the blue broken line is the sol–gel transition (Equation (36)). The dotted lines show the spinodal curve below which corresponds to the unstable region. We have five different phase separations: (1)–(5). See text for details.

Figure 5 shows the phase diagram on the temperature–concentration plane for $\alpha_{N}=1.5$. Further decreasing the value of the nematic parameter $a_{N}\left(=\nu /\chi \right)$, the binodal curve in the (I, Sol) region shifts upward, and we have the isotropic–isotropic phase separation (1) with a UCST. The closed circle shows the CP. At the TCP temperature (2), two isotropic phases and a gel phase coexist. In region (3), the phase separation between the isotropic sol and gel phases occurs. At the NIT temperature (4), we have the phase separation between the pure LC phase, where the I and N sol phases coexist, and the isotropic gel phase. Below the NIT temperature (5), we have the broad biphasic region between the nematic sol and isotropic gel phases, which appears in Figure 3.

**Figure 6:**
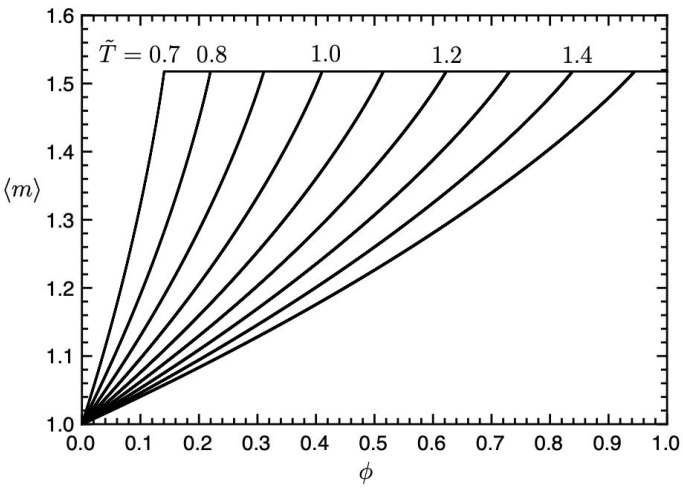
Average number 〈m〉 of *m*-clusters plotted against the concentration ϕ for various temperatures $\tilde{T}\left(=0.7,0.8,0.9,1.0,1.1,1.2,1.3,1.4,1.5\right)$ in Figure 5.

The number-average mean cluster size 〈m〉 of *m*-clusters can be calculated by $\langle m\rangle =\sum_{m=1}^{\infty }\phi_{m}/\sum \left(\phi_{m}/m\right)$. Figure 6 shows the average number 〈m〉 of *m*-clusters plotted against the concentration ϕ for various temperatures $\tilde{T}$ in Figure 5. The average number increases with the concentration ϕ and becomes a constant, ${\langle m\rangle }^{*}≃1.52$, at the sol–gel transition $\phi^{*}$. In the sol phase, the clusters are almost monomers and dimers. Due to the Flory–Huggins theory for non-reactive polymer blends, the CP concentration in the (I, Sol) region is given by

(38) $$\phi_{c}=\frac{\sqrt{n_{r}}}{\sqrt{n}+\sqrt{n_{r}}}.$$

When n=1 and $n_{r}=2$, we have $\phi_{c}=0.58$, which is almost the same as the CP concentration in Figure 5 because the number-average cluster size is a small number: 〈m〉≃1.3 in the sol region. On increasing the number *n* of the segments on the reactive molecules, the CP shifts to lower concentrations.

It has been observed that at-PS can gel in a series of solvent molecules, including toluene and tetrahydrofuran [18,19]. In our previous paper [22], we showed the comparison of the theoretical calculation with the observed phase diagram for the monodisperse at-PS in the solvent molecule CS${}_{2}$. The theory can properly describe the global characteristics of the phase behaviour of the thermoreversible gelation. Then, it is informative to calculate the phase diagram for the mixture of at-PS and LC, by using some numerical parameters which were used in the previous paper.

**Figure 7:**
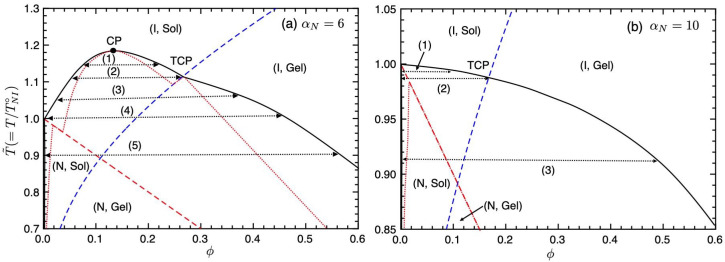
Phase diagrams on the temperature–concentration plane for the binary mixture of a reactive polymer and an LC for $\alpha_{N}=6$ (**a**) and $\alpha_{N}=10$ (**b**). The solid curve shows the binodal line, the red broken line is the NIT (Equation (35)), and the blue broken line is the sol–gel transition (Equation (36)). The dotted lines show the spinodal curve below which corresponds to the unstable region.

Figure 7 shows the calculated-phase diagrams on the temperature–concentration plane for the mixture of a reactive polymer (at-PS) and LC. We here use the numerical parameters as follows: $\gamma_{1}=4$, $\lambda_{0}=0.004$, n=100, f=15, $n_{r}=2$, $\alpha_{N}=6$ (a), and $\alpha_{N}=10$ (b). In Figure 7a, we predict CP, TCP, and five different phase separations (1)–(5) as shown in Figure 5. The binodal line of the (N, Sol) region shifts to lower concentrations because the number *n* of segments on the reactive polymer is large. The nematic phase almost consists of pure LCs, coexisting with the isotropic gel phase. The physical gelation of at-PS is universal, and so our results may be confirmed by the mixtures of at-PS and LC, such as 7CB or EBBA, etc. The UCST on Figure 7a depends on the nematic parameter $\alpha_{N}$. Figure 7b shows the phase diagram for $\alpha_{N}=10$. The CP disappears, and we have three different phase separations (1)–(3). Near $\tilde{T}=1$, we have the phase separation (1) between a nematic sol and an isotropic gel phase. At the TCP temperature (2), a pure nematic LC phase and isotropic sol, and gel phase coexist. Below TCP temperature (3), a pure nematic LC sol and an isotropic gel phase coexist. The spinodal region, or unstable region, appears only below the NIT line.

According to the Flory–Huggins theory for nonreactive polymer blends, the UCST, or CP temperature, in the (I, Sol) region is given by

(39) $$\chi_{c}=\frac{{\left(\sqrt{n}+\sqrt{n_{r}}\right)}^{2}}{2nn_{r}}.$$

We then obtain the UCST temperature

(40) $${\tilde{T}}_{c}=\frac{\tau_{c}}{\tau_{NI}^{\circ }}=\frac{1}{\chi_{c}\tau_{NI}^{\circ }}=\frac{9.1n}{\alpha_{N}{\left(\sqrt{n}+\sqrt{n_{r}}\right)}^{2}}.$$

(Note that Equation (40) is the UCST for nonreactive polymer blends.) Using Equation (36) and Equation (40), we can estimate the condition that satisfies ${\tilde{T}}_{c}>{\tilde{T}}_{SG}$ at the CP concentration $\phi_{c}$ as

(41) $$\alpha_{N}\leq \frac{-9.1n}{\gamma_{1}{\left(\sqrt{n}+\sqrt{n_{r}}\right)}^{2}}\ln\left(2\left(f-2\right)\lambda_{0}\phi_{c}\right).$$

Using the numerical parameters of Figure 7, we have the condition $\alpha_{N}\leq 7.7$ for ${\tilde{T}}_{c}>{\tilde{T}}_{SG}$. Figure 7a shows the result of $\alpha_{N}=6$ and we have ${\tilde{T}}_{c}>{\tilde{T}}_{SG}$. Of course, our system has 〈m〉≃1.2 in the (I, Sol) region as shown in Figure 5 and the UCST in Figure 7a is higher than ${\tilde{T}}_{c}=1.12$ obtained from Equation (40) because the UCST increases with increasing cluster size 〈m〉. Equation (41) is not an exact condition for ${\tilde{T}}_{c}>{\tilde{T}}_{SG}$ for our systems; however, it will be the index in order to consider experimental results and the meaning of the nematic parameter $\alpha_{N}$. For example, when the bonding energy parameter $\gamma_{1}$ is large, it is hard to be ${\tilde{T}}_{c}>{\tilde{T}}_{SG}$. On increasing the value of $\alpha_{N}$, the CP in Figure 7a disappears inside the (I, Gel) or (N, Sol) regions as shown in Figure 7b [10].

In our model, we neglect the nematic interactions between the LC and polymer network and then the (N, Gel) region becomes unstable on the phase diagrams. It has been reported these orientational-dependent interactions between unlike molecules also affect phase behaviours [11,31]. When the anisotropic coupling between the LC and polymer is strong, the NIT curve has a maximum as a function of ϕ on the temperature–concentration plane, and induced-nematic phases have been predicted [11]. In this case, the (N, Gel) region shifts to higher temperatures, and we can expect that a stable (N, Gel) region appears on the phase diagram. We also neglect the excluded volume interaction between rodlike molecules. In long rodlike molecular solutions, chimney-type phase separations between isotropic and nematic phases appear on the temperature–concentration plane [11]. Then, we can expect the interference between the chimney-type phase separation and the sol–gel transition. In this paper, we focus on nematic–isotropic phase transitions. Smectic A-isotropic and smectic A-nematic–isotropic phase transitions are also crucial to further fundamental research.

## 4. Conclusions

We predict the novel phase behaviours of thermoreversible gel-dispersed LCs. By changing temperature and concentration, the interference between sol–gel transition, nematic ordering, and phase separations causes new phenomena, such as TP, and TCP, and the coexistence between nematic sol and isotropic gel phases. Outside the binodal line of an isotropic phase in conventional polymer and LC mixtures, we have a miscible region where the mixture is in an isotropic fluid phase. However, our systems have the (I, Gel) region, where the mixtures exhibit an elastic response due to the gel. Thus, NIT and gelation are advantageous for integrating optical and mechanical devices for various chemical and biological sensing applications. We conclude that thermoreversible gel-dispersed LCs can be one of the new classes of temperature-sensitive PDLCs. These phase diagrams have not been observed yet, and we hope to achieve this with the experimental evidence of our results.

## Data Availability

The data presented in this study are available within the article.

## Appendix A. Derivation of Orientational Distribution Function

In this appendix, we derive the orientational distribution function $f_{d}\left(\theta \right)$, which is determined by minimising the free energy (Equation (4)). The minimum is found by introducing a Lagrange multiplier Λ and solving

(A1) $$\frac{\delta }{\delta f_{d}\left(\theta \right)}\left[F_{nem}\left(f_{d}\left(\theta \right)\right)-\Lambda \int f_{d}\left(\theta \right)d\Omega \right]=0.$$

with the condition Equation (5). This yields

(A2) $$\int d\Omega \left(\frac{\phi_{r}}{n_{r}}\ln4\pi f_{d}\left(\theta \right)-\nu \phi_{r}^{2}SP_{2}\left(\cos\theta \right)-\Lambda \right)=0,$$

and leads to

(A3) $$\ln4\pi f_{d}\left(\theta \right)=n_{r}\nu \phi_{r}SP_{2}\left(\cos\theta \right)+\Lambda .$$

Then we obtain the distribution function (Equation (7)):

(A4) $$f_{d}\left(\theta \right)=\frac{1}{4\pi }\exp(\Lambda +n_{r}\nu \phi_{r}SP_{2}\left(\cos\theta \right))=\frac{1}{Z_{0}}\exp(n_{r}\nu \phi_{r}SP_{2}\left(\cos\theta \right)).$$

where the constant $Z_{0}(=4\pi /\exp\left(\Lambda \right)$) is determined by the normalisation condition (Equation (5)).

## Appendix B. Application of Stockmayer’s Distribution Function

In this appendix, we derive the sum of the infinite series $\sum \phi_{m}$ explicitly [27]. Using Equation (21), the total volume fraction of reactive molecules is given by

(A5) $$\phi =\frac{{\left(1-p\right)}^{2}}{pe}\sum_{m=1}^{\infty }\frac{(fm-m)!}{m!(fm-2m+2)!}x^{m}.$$

where $x=\lambda \phi_{1}$. We here define the function $S_{i}\left(x\right)$:

(A6) $$S_{i}\left(x\right)=\sum_{m=1}^{\infty }\frac{m^{i}\left(fm-m\right)!}{m!(fm-2m+2)!}x^{m},$$

for i=0,1,2. For f=2 the results are well known. Using the parameter α defined as $x=\alpha {\left(1-\alpha \right)}^{f-2}$, we obtain

(A7) $$S_{0}\left(x\right)=\frac{\alpha (1-f\alpha /2)}{{\left(1-\alpha \right)}^{2}f},$$

(A8) $$S_{1}\left(x\right)=\frac{\alpha }{{\left(1-\alpha \right)}^{2}f},$$

and

(A9) $$S_{2}\left(x\right)=\frac{\alpha (1+\alpha )}{{\left(1-\alpha \right)}^{2}f\left(1-\left(f-1\right)\alpha \right)}.$$

Substituting Equation (A7) into Equation (A5), we obtain

(A10) $$\lambda \phi =\frac{\alpha (1-f\alpha /2)}{{\left(1-\alpha \right)}^{2}},$$

where $\lambda =efp/{\left(1-p\right)}^{2}$. The solution is given by

(A11) $$\alpha =\frac{1+2y-a(y)}{f+2y},$$

where we define $a\left(y\right)=\sqrt{1-2y(f-2)}$ and y=λϕ for y≤1/(2(f−2)). When y=1/(2(f−2)), we have a critical value of $\alpha_{c}=1/\left(f-1\right)$ and the sums $S_{0}$ and $S_{1}$ converge, but $S_{2}$ diverges. The value of $\alpha_{c}$ yields the sol–gel transition point (Equation (22)). Substituting Equation (A11) into $x=\alpha {\left(1-\alpha \right)}^{f-2}$, we obtain the volume fraction $\phi_{1}$ as a function of ϕ:

(A12) $$\lambda \phi_{1}=\frac{\left(1+2y-a\left(y\right)\right){\left(f-1+a\left(y\right)\right)}^{f-2}}{{\left(f+2y\right)}^{f-1}},$$

When λ is given as a function of *T* at a given ϕ, we obtain the value of α from Equation (A11) and the value of $\phi_{1}$ from Equation (A12). The function κ(ϕ) in Equation (31) is given by

(A13) $$\kappa \left(\phi \right)=\phi \left(\frac{d\ln\phi_{1}}{d\phi }\right)=\frac{1}{2(f+2y)}\left(f(1+a(y))-2y(f-2)\right).$$

For a gel phase, the value of $\phi_{1}$ is a constant $\phi_{1}^{*}={\left(f-2\right)}^{f-2}/\left(\lambda {\left(f-1\right)}^{f-1}\right)$ and we have κ(ϕ)=0.
